# *Leishmania* Specific CD4 T Cells Release IFNγ That Limits Parasite Replication in Patients with Visceral Leishmaniasis

**DOI:** 10.1371/journal.pntd.0003198

**Published:** 2014-10-02

**Authors:** Rajiv Kumar, Neetu Singh, Shalini Gautam, Om Prakash Singh, Kamlesh Gidwani, Madhukar Rai, David Sacks, Shyam Sundar, Susanne Nylén

**Affiliations:** 1 Institute of Medical Sciences, Banaras Hindu University, Varanasi, Uttar Pradesh, India; 2 Department of Immunology and Infection, Queensland Institute of Medical Research, Herston, Queensland, Australia; 3 Department of Biotechnology, University of Turku, Turku, Finland; 4 National Institute of Allergy and Infectious Diseases, National Institutes of Health, Bethesda, Maryland, United States of America; 5 Karolinska Institutet, Department of Microbiology Tumor and Cell Biology, Stockholm, Sweden; Hospital Universitário, Brazil

## Abstract

Visceral leishmaniasis (VL) is associated with increased circulating levels of multiple pro-inflammatory cytokines and chemokines, including IL-12, IFNγ, and TNFα, and elevated expression of IFNγ mRNA in lesional tissue such as the spleen and bone marrow. However, an immunological feature of VL patients is that their peripheral blood mononuclear cells (PBMCs) typically fail to respond to stimulation with leishmanial antigen. Unexpectedly, it was recently shown that *Leishmania* specific IFNγ, can readily be detected when a whole blood stimulation assay (WBA) is used. We sought to define the conditions that permit whole blood cells to respond to antigen stimulation, and clarify the biological role of the IFNγ found to be released by cells from VL patients. CD4+ T cells were found to be crucial for and the main source of the IFNγ production in *Leishmania* stimulated whole blood (WB) cultures. Complement, antibodies and red blood cells present in whole blood do not play a significant role in the IFNγ response. The IFNγ production was reduced by blockade of human leukocyte antigen (HLA)-DR, indicating that the response to leishmanial antigens observed in WB of active VL patients is a classical HLA- T cell receptor (TCR) driven reaction. Most importantly, blockade of IFNγ in *ex-vivo* splenic aspirate cultures demonstrated that despite the progressive nature of their disease, the endogenous IFNγ produced in patients with active VL serves to limit parasite growth.

## Author Summary

Our research aims to understand the immune failure underlying progression of human visceral leishmaniasis (VL). A key immunological feature of VL patients is that their peripheral blood mononuclear cells (PBMCs) do not respond to stimulation with leishmanial antigen. Surprisingly, when employing a whole blood assay we discovered significant levels of IFNγ in response to soluble *Leishmania donovani* antigen (WBA) in VL patients. We were interested to understand the relevance of the IFNγ to the anti-parasitic response. Animal models and *in vitro* studies have shown that IFNγ is a key effector cytokine required for control of the infection, however, the role of endogenous IFNγ in control of parasites in VL patients, has not been demonstrated. Our results show that CD4 cells were required for and were the source of *Leishmania* specific IFNγ in WBA of VL patients. Optimal IFNγ response required interaction with HLA-DR, supporting that VL is not due to an intrinsic Th1 response defect *per se*. The *Leishmania* driven IFNγ appears to limit parasite growth in patients with active VL, since blockade of IFNγ *ex-vivo* in splenic aspirate cultures enhanced parasite survival. This suggests that IFNγ may have been prematurely dismissed as an adjunct therapy in treatment of VL.

## Introduction

Visceral leishmaniasis is a chronic disease caused by the protozoan parasites *Leishmania donovani* and *Leishmania infantum/chagasi. Leishmania* are transmitted by the bite of phlebotomine sand flies, and replicate within macrophages of their mammalian hosts. In VL, the target organs are chiefly the liver and the spleen. The disease is characterized by prolonged fever, spleno-hepatomegaly, wasting, hypergammaglobulinemia, pancytopenia and almost always leads to death if left untreated.

Based on experimental models, acquired resistance against *Leishmania* infection requires the development of a Th1 type immune response, characterized by IL-12 production by antigen presenting cells (APC) and IFNγ production by T cells [1], [2]. IFNγ is a key effector cytokine required for activation of infected macrophages for killing (reviewed by Kima and Soong [3]). Patients with active VL have depressed cell-mediated immune responses, reflected by the failure of their peripheral blood mononuclear cells (PBMCs) to proliferate and/or to produce IFNγ in response to stimulation with *Leishmania* antigens, while their ability to respond to polyclonal stimulation or other antigens, such as the purified protein derivative of *Mycobacterium tuberculosis* (PPD), remains relatively intact [4], [5]. The absence of antigen specific responses is thought to underlie the disease progression. Paradoxically, the acute phase of VL is associated with elevated expression of IFNγ mRNA in lesional tissue, such as the spleen and bone marrow, as well as increased circulating levels of multiple pro-inflammatory cytokines and chemokines, including IL-12, IFNγ and TNFα [4], [6]. These results imply that the failure to respond to *Leishmania* antigen stimulation observed in VL patients is not due to a defect in the ability to mount protective Th1 responses *per se*, but rather to induction of suppressive factors, e.g. IL-10, resulting in unresponsiveness of infected macrophages to activation signals [7].

Studying immunological aspects of human VL has been severely hampered by the inability to measure antigen specific responses, including IL-10, using PBMC. The discovery of antigen specific cytokine responses following stimulation of whole blood (WB) [8] showed that VL patients are not void of *Leishmania* specific IFNγ responses, findings that could be reconciled with the elevated levels of IFNγ mRNA and circulating cytokines detected in active VL patients. Subsequent studies reported that the whole blood assay (WBA) could also be used to detect antigen-specific IL-10 responses [9], [10]. Thus, the WBA has opened up new possibilities for research aimed at understanding immunological determinants of the disease [8], [9], [10], [11].

We sought to define the requirements for IFNγ production seen using the WBA, and determine if the IFNγ had a biological function in patients with active VL. We show that CD4+ T cells produce *Leishmania* specific IFNγ in WB cultures. The responses to stimulation with *Leishmania* antigen observed in WB cultures of active VL patients occurred in the absence of complement, antibodies or cytokines present in serum of VL patients. Employing a splenic aspirate (SA) culture technique, as previously described [11], we show that IFNγ neutralization promotes parasite growth in active VL cases *ex-vivo*. These findings demonstrate that the elevated levels of IFNγ in patients with active VL serve to limit parasite replication and suggest that therapeutic administration of IFNγ may still hold potential.

## Materials and Methods

### Study groups

All VL patients presented with clinical symptoms of kala-azar at the Kala-azar Medical Research Center (KAMRC), Muzaffarpur, Bihar, India, and were confirmed to be VL positive by detection of amastigotes in splenic aspirates and/or by detection of antibodies against the recombinant antigen, K39. Venous blood and/or splenic aspirates (SA) samples collected from 84 (33 female and 51 male) patients with active VL were included in this study. All patients were treated with Amphotericin B and eventually cured disease. Aggregate clinical data of active VL patients are presented in Table 1.

**Table 1:** Aggregate clinical data for VL patients.

N	84
Age (years)	29.45±17.6
Sex (M/F)	51/33
Duration of illness (days)	37.31±35.29 (30)
WBC counts, pre treatment	3332±1183 (3100)
WBC counts, post treatment^a^	6795±2887 (6300)
Spleen Size (cm), pre treatment	4.43±2.82 (4)
Spleen Size (cm), post treatment^a^	0.81±1.42 (0)
Splenic Score^b^	2.37±1.11^c^

Median values are given within brackets.

a Post treatment values are from day 15 or day 30 post treatment.

b Scoring of parasite load is on a logarithmic scale from 1 to 6, were 0 is no parasites per 1000 microscopic fields (1000×), 1 is 1–10 parasites per 1000 fields, and 6 is >100 parasites per field. ND = not done; NA = not applicable.

c Splenic scores presented are only based on patients used to assess the *ex-vivo* effect of IFNγ blockade.

### Ethics statement

The use of human subjects followed recommendations outlined in the Helsinki declaration. Informed written consent was obtained from all participants and/or their legal guardian when under 18 years of age. All human samples were coded an analysed anonymously. Ethical approval (Dean/2008-09/314, Dean/2012-2013/89) was obtained from the ethical review board of Banaras Hindu University (BHU), Varanasi, India.

### Stimulation of whole blood (WB)

Whole blood (WB) was cultured using a volume of 0.5–1 ml blood per culture condition in round bottom 5 ml polypropylene tubes. For stimulation the samples were treated with SLA (10 µg/ml). Control samples were treated with PBS. In some assays PHA (10 µg/ml) or *Staphylococcus* enterotoxin B, SEB, (5 µg/ml) was used as positive controls (not shown). Samples were incubated for 37°C in the presence of 5% CO_2_ for 24 hours if not otherwise indicated in figure text.

To block HLA-TCR interaction 20 µg/ml anti-HLA-DR, clone 243, or isotype control IgG2a, clone MOPC-173, both ultra-LEAF purified (BioLegend, US) were added to the cultures simultaneously with antigen.

### Inactivation of complement and replacement of plasma

To test if complement, antibody and/or other proteins present in plasma, but removed during purification of PBMC, affected SLA induced IFNγ production we replaced the plasma in the WB samples. In brief, total blood cells were pelleted by centrifugation (500 g, 10 minutes, 18°C), the plasma was removed and blood was washed twice with PBS. To determine if complement affected the response, the plasma was heat inactivated [12] at 56°C for 30 minutes and added back to the autologous sample to restore the original blood volume. Alternatively, the plasma was replaced with heat inactivated fetal calf serum (HI-FCS).

### Depletion and isolation of cell subsets

To determine the effects of different cell populations on SLA induced IFNγ production we used magnetic beads and columns designed for the isolation/depletion of CD4 and CD8 cell subsets from whole blood as per manufacturer protocol (Whole Blood Column kit, Milteny Biotec). To control for the effect and spontaneous uptake of magnetic beads [13] we used anti-FITC beads (Milteny Biotech) as control. Whole blood and whole blood depleted of the cell subsets of interest were subsequently stimulated as described above. In these assays the patient plasma was replaced with HI-FCS prior to incubation with whole blood beads.

### Lysis of red blood cells (RBC)

The influence of RBC on SLA induced IFNγ was tested by lysis of RBC. Briefly, the total blood cells were centrifuged (500 g, 10 minutes, 18°C), followed by removal of plasma (see above). The cell pellet was resuspended in 5 ml hypotonic saline (0.6% NaCl) solution/1 ml blood for 20–30 seconds to lyse RBC. To stop the lysis an equal volume of hypertonic solution (1.6% NaCl) was added. The tube was filled with PBS and the non-lysed cells were pelleted and resuspended in autologous plasma to reconstitute the original volume, and stimulated as described above.

### Culture of splenic aspirate (SA) cells

Splenic needle aspirates were collected for diagnostic purposes before treatment of VL. Approximately 100 µl SA was obtained by fine needle biopsy, following preparation of smears for diagnostic purpose, the residual cells were placed directly in 1 ml RPMI supplemented with 10% heat-inactivated fetal calf serum (HI-FCS) 200 mM Streptomycin and 100 U/ml penicillin (C-RPMI) and 5 U/ml heparin. Samples were transported to the laboratory at BHU maintaining a temperature of 4–8°C. All samples were processed within 24 h of collection. For stimulation, the SA divided into two equal parts and treated with SLA (10 µg/ml) as done for the in the WBA (described above).

For baseline quantification of amastigotes by limiting dilution, 150 µl SA suspension was directly plated in a 96-well and serially diluted by transfer of 50 µl SA onto biphasic medium of 50 µl blood agar overlaid by 100 µl of M199/C, as previously described [14]. The remaining SA suspension was seeded into 96 well-culture plates (250 µl/well). Monoclonal antibody against human IFN-γ, clone 25723 (R&D Systems) or control IgG2b clone 20116 (R&D Systems) were each added to a final concentration of 20 µg/ml. The SA was incubated for 3 days at 37°C in 5% CO_2_, the supernatants were collected for cytokine assessment and the removed volume replaced by C-M199 medium, prepared as previously described [15]. From the SA culture 150 µl was transferred into a 96-well plate for estimation of parasite load by limiting dilution as described above. The number of viable parasites was determined from the highest dilution at which promastigotes could be grown out after 7 to14 days of incubation at 25°C.

For comparison with the WB, SA suspension was divided in two parts, stimulated with SLA (10 µg/ml) or with PBS and incubated for 24 hours at 37°C in 5% CO_2_, where after the supernatant was collected for cytokine assessment.

### Cytokine analysis

Following 24 hours of stimulation (if not otherwise indicated) IFNγ and IL-10 were measured in culture supernatants by ELISA. ELISA was performed as per manufacture instruction. For detection of IFNγ the ELISA Max Deluxe set (BioLegend) or the QuantiFeron kit (Cellestis, Australia) were used. IL-10 was measured using matched antibody pair kits from BD Pharmingen. All values calculated from standard curve over or equal to zero were considered in statistical analysis. Negative values were assigned the value zero.

### Flow cytometry (FACS) assessment of intracellular cytokine

To determine the cellular source/s of cytokines in the WBA, the cultures were stimulated for 16–24 hours. To block cytokine secretion cultures were for the last 6–8 hours of stimulation treated with GolgiStop (BD Biosciences) according to manufactures instructions. Following lysis of RBC using BD RBC lysis buffer (BD Biosciences), cells were surface stained using combinations of FITC, PE and PerCP/PE-Cy5 conjugated antibodies directed to CD3 (Clone UCHT1), CD4 or CD8 (all from BD Biosciences). Surface stained cells were fixed and permeabilized using BD Cytofix/Cytoperm, as per manufactures instruction, washed in permeabilization buffer (BD) and stained for presence of intracellular IFNγ and IL-10 using APC and PE conjugated antibodies (both from Pharmingen) respectively. Following intra cellular staining (ICS), samples were acquired on FACSort (BD Biosciences) and analyzed using CellQuest Pro (BD) or FlowJo (Treestar) software. Analysis was done on cells gated as viable lymphocytes based on their forward–side scatter. SEB (10 µg/ml) stimulated samples were used as positive control for ICS (not shown).

### Statistical analyses

Statistical analyses were done using PRISM5 (GraphPad Software). Different treatments using the same donor samples were compared by the Wilcoxon signed rank test for paired samples. Correlation between results was determined using Spearman-test for non-parametric correlations. Differences with P-values<0.05 were considered as significant. Outliers (donors with extreme values in one or more of the test conditions) were removed from data sets after being defined as outlier using GraphPad on-line Grubb's test for outliers.

## Results

### Kinetics of *Leishmania* specific IFNγ secretion in whole blood cultures of patients with active VL

**Figure 1:**
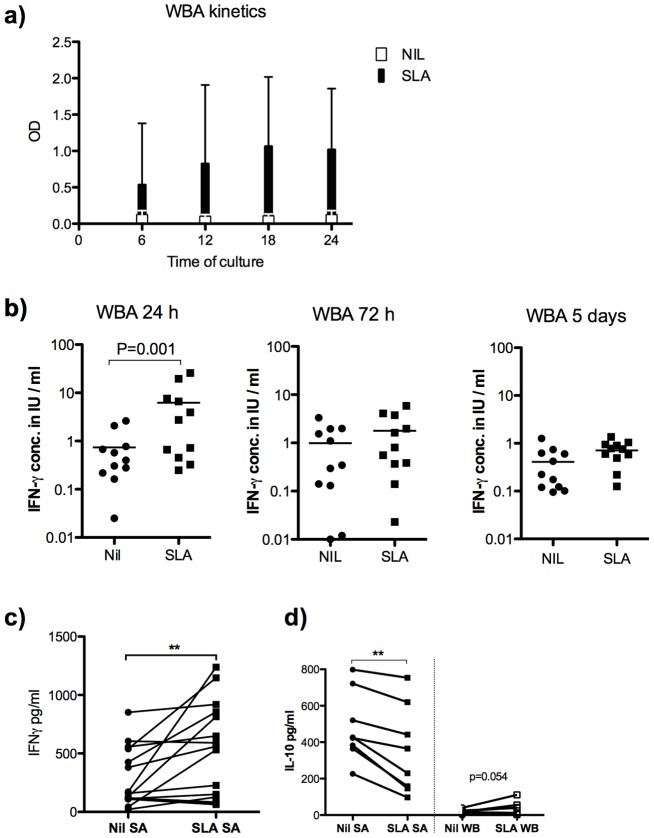
Induction of IFNγ in SLA stimulated whole blood and splenic aspirates from patients with active VL. a) IFNγ in SLA stimulated (black bars) and unstimulated (white bars) WB during the first 24 hours of culture and b) IFNγ production (measured in units by QuantiFeron kit) in culture supernatants from SLA stimulated WB after 24 hours, 72 hours and 5 days incubation at 37°C, 5%CO_2_. c) IFNγ in 24 hour cultures of SLA stimulated and unstimulated SA from patients with active VL (n = 15) and d) IL-10 in 24-hour SLA stimulated and unstimulated SA (left) and WB (right), n = 8. Results show in a mean OD+SD of the samples tested, in b–d one dot represents one sample. Stimulated and unstimulated samples were compared by Wilcoxon matched paired test and statistical significances are indicated with *** p<0.001, ** p<0.01 and * p<0.05.

The whole blood Quantiferon assay (WBA) was originally designed as a tool for diagnosis of tuberculosis, and detects cytokine (IFNγ) concentrations in plasma supernatants after 16–24 hours of incubation with antigen. To determine the kinetics of the WB responses in VL patients we measured secreted cytokines in supernatants after 6 hours to five days of stimulation with soluble *Leishmania* antigen (SLA). The induction of IFNγ was rapid and observed in supernatants already 6 hours after stimulation, reaching a plateau at 18–24 hours (Figure 1a, b). Antigen-induced IFNγ was not detected in WB cultures following 72 hours culture or more (figure 1b). We conclude that the IFNγ response seen in the WB cultures is rapid and short lived. For practical reasons stimulation times of 24 hours were used in subsequent assays if not otherwise indicated.

We further tested if antigens specific responses could be detected in short-term (24 hr) splenic aspirate (SA) cultures. In line with the observations made using the WBA, an increase in IFNγ was observed in supernatants of 73% of SA cultures following stimulation with SLA, indicating that antigen specific cells are present at the site of infection (figure 1c). In contrast to the SLA stimulated WB cultures where IL-10 tended to be induced [9], [10], IL-10 levels dropped in SA cultures following SLA stimulation (figure 1d).

### The WB response was not affected by serum complement inactivation, replacement of autologous plasma, or red blood cell lysis

**Figure 2:**
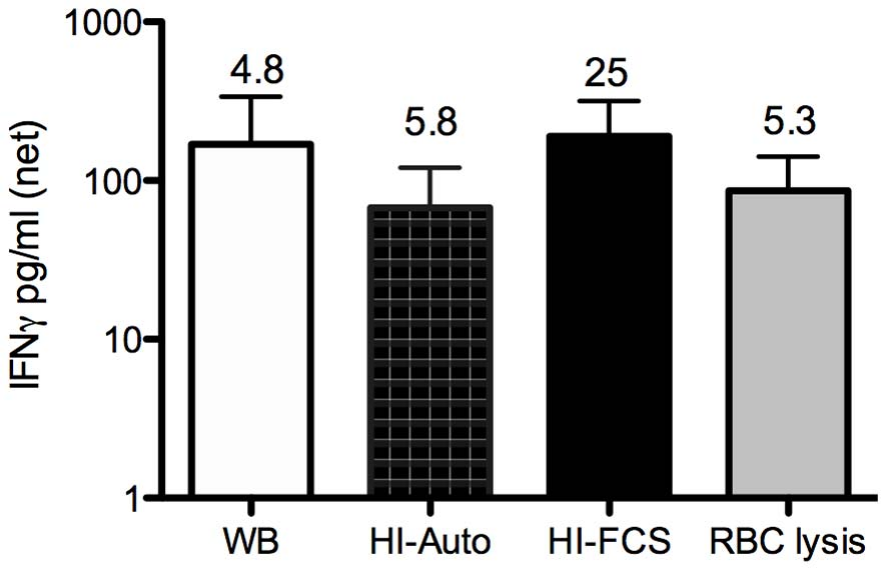
SLA induced IFNγ secretion in WB of active VL patients is not dependent on plasma proteins or RBC. IFNγ in SLA stimulated WB from active VL patients (n = 8/9) following replacement of plasma or lysis or RBC as indicated (WB = whole blood untouched; HI-Auto = replacement with heat inactivated [12] autologous plasma; HI-FCS = replacement with HI-FCS). Net responses (stimulated minus unstimulated)+SD are shown. The stimulation indices (stimulated/unstimulated) are indicated above each bar to as an additional comparison. Comparisons between treatments were made using Wilcoxon matched paired test; there were no significant differences between the different culture conditions.

The immune system of patients with VL is highly activated. We considered the possibility that other blood cell or serum components that are removed in the process of PBMC purification could be required for the *Leishmania* specific WB response. To address the effect of plasma components we replaced the plasma with i) autologous heat-inactivated plasma, to determine the role of complement, or ii) heat inactivated fetal calf serum (HI-FCS), to remove antibodies, complement, or other serum factors such as cytokines that may be elevated in VL. To address if RBC were important, we lysed the RBC using hypotonic treatment. None of these treatments affected the net production of IFNγ measured using the WBA (figure 2), indicating that complement, antibodies, cytokines, or RBC are not important for the observed SLA induced IFNγ production in WB. Indeed, removal of autologous plasma with HI-FCS potentiated the SLA induced response (figure 2). The replacement of plasma with FCS was subsequently employed in some of the assays that followed.

### CD4 T cells are the source of IFNγ in the WB response to SLA in patients with active VL

**Figure 3:**
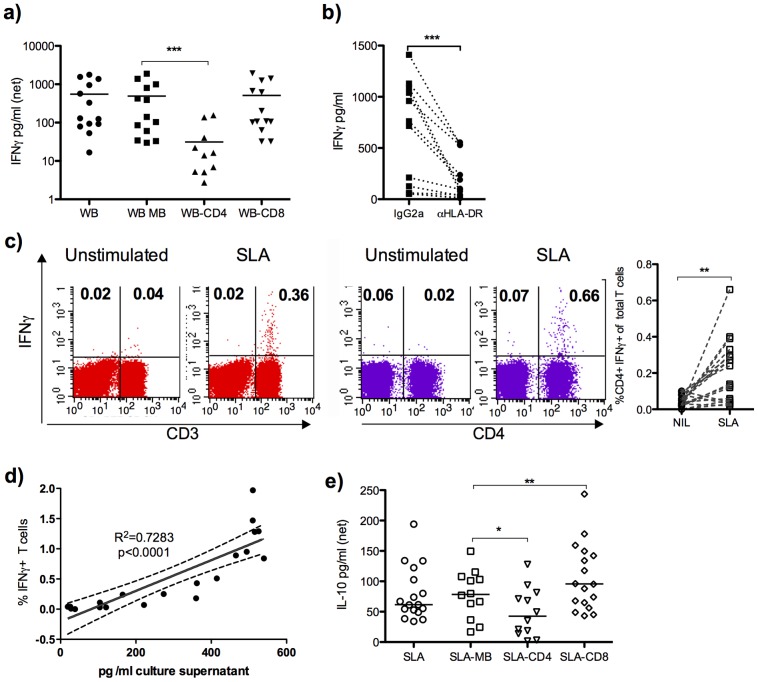
CD4 cells are necessary for and the source of IFNγ in SLA stimulated WB from patients with active VL. IFNγ production in SLA stimulated WB cultures of active VL patients following depletion of a) CD4 and CD8 positive cells. b) Effect of pan-HLA-DR blockade on SLA induced IFNγ, compared to IgG2a isotype control treatment c) Intra cellular IFNγ lymphocytes, following SLA stimulation of WB culture. The dot-plots to the left (red) show IFNγ production in total gated lymphocytes and the dot-plots to the right (purple) show SLA induced IFNγ in gated CD3+ cells. In the graph the frequencies of IFNγ+ CD4+ T cells are shown; combined results of three different experiments. d) Correlation between percentages of IFNγ positive cells in gated CD3+ lymphocytes as determined by ICS and IFNγ levels in WB supernatants following 24 hours of SLA stimulation. e) IL-10 levels in SLA stimulated WB supernatants following removal of CD4 and CD8 positive cells. Plasma was replaced with HI-FCS prior to incubation with MicroBeads or HLA-DR blockade, MB = mock treatment with magnetic beads directed against FITC. Each dot represents one sample. Net SLA response (e.g. stimulated minus unstimulated) samples are shown, if not otherwise indicated. Comparison between treatments was made using Wilcoxon matched paired test and statistical significances are indicated with *** p<0.001, ** p<0.01 and * p<0.05.

Understanding the cellular source/s of IFNγ in the WB is critical to our reinterpretation of the immunologic defects in kala-azar. To determine the cellular requirements for IFNγ production we removed various subsets from whole blood of VL patients prior to stimulation with SLA. Removal of CD4 cells caused a substantial loss of SLA induced IFNγ in WB cultures, while removal of CD8 cells had no effect (figure 3a). Blockade of HLA using a pan-HLA-DR antibody caused a significant loss of SLA induced IFNγ (figure 3b). This suggests that the IFNγ response induced by SLA stimulation depends on HLA-TCR interaction. Three out of the 12 patient samples in which the effect of HLA-DR blockade was evaluated had low IFNγ responses to SLA (<100 pg/ml). To confirm CD4 T cells as the source of IFNγ in WB, we assessed intracellular IFNγ by FACS. SLA induced IFNγ was only observed in the CD3+ population (all events considered). Figure 3c shows that the IFNγ is produced by CD3+CD4+ cells, while figure 3d shows that there is a strong correlation between the frequency of IFNγ positive T cells (CD3+) and the IFNγ measured in WB culture supernatants by ELISA. IFNγ was not detected in the CD3+CD8+ population following SLA stimulation and almost all cells producing IFNγ following SLA stimulation were CD3+CD8− (not shown).

To test if neutrophils contributed to the IFNγ responses CD15+ cells were removed using depletion beads, this caused a partial though significant loss of SLA induced IFNγ (figure S1), which may indicate an involvement of neutrophils in the observed SLA response, but since CD15 can be expressed on other cells, i.e. monocyte, we cannot exclude that the effect seen is due to removal of these cells.

IL-10 can be induced in stimulated WB from VL patients, albeit at low levels. Removal of CD4 cells caused a small but significant reduction of the amount of detectable IL-10 in SLA stimulated WB (figure 3e), indicating that CD4+ and other cells are sources of antigen-specific IL-10 in VL patients. CD8 cells do not appear to contribute to SLA induced IL-10 response, and their removal caused a slight enhancement of this response (Figure 3e). The source of SLA induced IL-10 could not be confirmed by intracellular staining as the number IL-10 positive cells was below the limit of reliable detection.

### Endogenous IFNγ limit parasite replication in the spleen of visceral leishmaniasis patients

**Figure 4:**
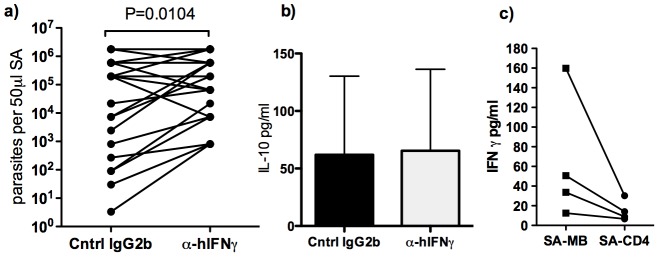
Endogenous IFNγ limit parasite replication. a) Parasite load in *ex-vivo* SA culture of VL patients (n = 31) treated with neutralizing anti-human-IFNγ antibodies or isotype control IgG2b antibodies for three days. Parasite load was determined by limiting dilution on NNN-blood agar medium. b) IL-10 levels in SA culture supernatants (n = 10), treated as described in a. c) Effect of CD4 depletion on spontaneous IFNγ production in SA-biopsy cultures. Each dot represents one sample, paired samples connected by a line. Comparison between treatments was made using Wilcoxon matched paired test and statistical significances are indicated in the graphs when P<0.05.

In experimental models it is well established that IFNγ mediates control of parasite replication [16] and that lack of IFNγ signalling causes disease progression [17], [18]. The same protective function is assumed in humans, but the direct proof that IFNγ controls parasite replication in human VL is lacking. To test if the endogenous IFNγ, which we now know to be elevated during active disease, plays a role in parasite control, we treated *ex-vivo* SA cultures with neutralizing antibodies against human IFNγ followed by assessment of parasite growth, as previously described in assays designed to test the function of endogenous IL-10 [11]. Following neutralization of IFNγ, the parasite load in SA increased in 19/31 (61%), was unchanged in 8/31 (26%) and decreased in 4/31 (13%) samples (figure 4a). The IL-10 levels in the SA supernatants were not affected by neutralization of IFNγ (figure 4b), suggesting that the inhibitory effect of IL-10 on parasite killing does not completely abolish the parasite-controlling effects of endogenous IFNγ. The background levels of IFNγ detectable in *ex vivo* SA cultures were significantly reduced when CD4 cells were removed (figure 4c), indicating that CD4 cells are needed for the splenic IFNγ production.

## Discussion

In the search for markers of *L. donovani* infection, epidemiological studies utilising a WBA revealed *Leishmania* specific IFNγ responses, long considered absent, in patients with active VL [8]. The goals of the current study were to validate the prior WBA results, to reveal the conditions required for SLA induced IFNγ secretion by WB and to determine if the IFNγ seen in patients with active disease functions to limit the infection.

Whole blood contains cell populations, proteins, lipids and sugars that are largely removed when PBMC are purified. To test if such components were required for the antigen specific response we deprived WB cultures of RBC, plasma and complement. We found that replacement of autologous plasma and RBC lysis had no effect on the SLA induced IFNγ response. By contrast, removal of CD4+ cells revealed these cells to be the main source of antigen specific IFNγ secretion in the WB cultures, a finding that was substantiated by direct intracellular staining. In line with previous observation CD8 T cells were not found to contribute to SLA responses in patients with active VL [19].

Removal of CD15+ cells also reduced the IFNγ levels detectable in the SLA stimulated WB. CD15 (Lewis X) is a carbohydrate adhesion molecule primarily expressed on mature neutrophils in blood, but is also present on a subset of monocytes [20]. The decline in IFNγ levels following CD15 depletion may thus be explained by a reduction of APCs required for the T cell response, but could also imply that neutrophils contribute to the response. By contrast, Abebe *et al.* have proposed, based on the observation that VL patients have more CD15+ and higher content of arginase expressing CD15+ cells pre compared to post treatment patients or endemic controls, that neutrophils contribute to the unresponsiveness of VL PBMC [12]. Neutrophil inhibition of the antigen-specific IFNγ response in VL patients is not supported by the data presented here, where a reduction in IFNγ secretion by WB cells was observed following CD15 depletion.

The detection of IFNγ responses in stimulated splenic aspirate cells (figure 1c) indicates that antigen specific and responsive cells are present at the site of infection. Depletion of CD4 cells from *ex vivo* SA cultures support these cells as the source of IFNγ at the site of infection. In contrast to the WB, where IL-10 was also induced following SLA stimulation, IL-10 levels decreased in SA following SLA stimulation (figure 1d). More critically, we show that the endogenous IFNγ produced by splenic cells is biologically active and served to limit parasite growth in the SA cultures from the majority of VL patients, as shown by the increase in parasite numbers after IFNγ neutralization *ex-vivo*. The lack of effect of the IFNγ neutralization on parasite growth observed in some samples can be attributed to the nature of the SA. The sampling is done blind and the aspirates may vary in red and white blood cell content as well as the extent of disruption of infected cells, resulting in extracellular amastigotes that will be unaffected by the level of IFNγ released. The treatment with anti-IFNγ-antibodies did not affect the IL-10 levels detected in the SA supernatants (figure 4), suggesting that the inhibitory effects of IFNγ on parasite survival and growth occurs even in the presence of high levels of IL-10. The IFNγ response we detect in active cases, while functional, is clearly not a sufficient condition for cure, as the patients would succumb to the disease without treatment. We propose that fragility and/or short life span of these cells may limit their ability to mediate a fully curative response, although other factors, in particular IL-10, are clearly involved [11].

Our data suggest that even in untreated patients, their disease progression would be far worse in the absence of the endogenous IFNγ that they produce. Notably, there are patients whose cellular responses cannot be detected even when using the WBA. While not directly reflected in the clinical parameters (i.e. blood chemistry), these patients may have progressed further in the disease and lost the responding population. It may be noted that there was a negative correlation between SLA induced IFNγ response in WBA and parasite load in blood (Spearman r = −0.66; p = 0.004, n = 17), which indicates that the WB SLA response to a degree may reflect the severity of disease. Genetic or acquired defects in their ability to mount Th1 responses to *Leishmania* may also underlie the lack of response in some patients. We found that the SLA induced IFNγ response involved HLA-DR interaction as treatment with HLA-DR blocking antibody reduced the IFNγ levels in all donors tested (figure 3b), with an average decrease of 70% compared to control antibody treatment. The partial effect observed may be explained by utilization of HLA-DQ in the presentation of leishmanial antigens to T cells. While HLA-DR together with it's peptide is the classical ligand for T cells recognizing foreign antigens, HLA-DQ may also present peptides from pathogens and initiate T cells responses. The role of HLA molecules on WB SLA responses are of interest since risk alleles for development of VL were recently identified within in the MHC class II region [21]. The influence of allelic differences and role of different MHC molecules in the ability to drive *Leishmania* specific responses in the WB culture are under current investigation.

The functional Th1 response in active VL patients may also be highly relevant to their response to treatment. *L. donovani* infection in T cell deficient mice revealed a clear role for antigen specific T cells in the curative response to pentavalent antimony [22]. Our findings reinforce the rationale for the prior VL treatment trials carried out in the 1990s involving recombinant IFNγ, indicating that monotherapy could be beneficial [23], [24]. The lack of response to monotherapy in some patients and the absence of a long-lasting therapeutic effect, as well as the limited success as adjunct therapy with sodium stibogluconate [25], discouraged further trials. Our present and more recent studies suggest that antigen-specific IFNγ production may in some patients not be the limiting factor in their non-curative response.

In summary, our data support the notion that disease progression in VL is not due to a complete failure in Th1 development. Our findings make clear that WB cultures may allow detection of functionally relevant immune responses not seen using PBMC. Most patients with VL have antigen specific CD4 T cells capable of secreting IFNγ both in the blood and at the site of infection - the spleen. We further show that the IFNγ produced by VL patients play a role in limiting parasite growth.

## Supporting Information

Figure S1 **CD15 cells contribute to SLA responses in WB cultures of VL patients.** Effect of CD15 depletion (MACS, Miltenyi) on SLA driven IFN-γ response in WB cultures from VL patients. Net responses (SLA stimulated minus unstimulated are shown). Comparison between treatments (CD15 beads or control beads = MB) was made using Wilcoxon matched paired test, and statistical significance is indicated with P-value.
(TIF)

## References

[1] Heinzel FP, Sadick MD, Holaday BJ, Coffman RL, Locksley RM (1989) Reciprocal expression of interferon gamma or interleukin 4 during the resolution or progression of murine leishmaniasis. Evidence for expansion of distinct helper T cell subsets. J Exp Med 169: 59–72. 2521244 10.1084/jem.169.1.59 PMC2189187

[2] Scott P, Pearce E, Cheever AW, Coffman RL, Sher A (1989) Role of cytokines and CD4+ T-cell subsets in the regulation of parasite immunity and disease. Immunol Rev 112: 161–182. 2575073 10.1111/j.1600-065x.1989.tb00557.x

[3] Kima PE, Soong L (2013) Interferon gamma in leishmaniasis. Front Immunol 4: 156. 23801993 10.3389/fimmu.2013.00156 PMC3685816

[4] Nylen S, Maurya R, Eidsmo L, Manandhar KD, Sundar S, et al (2007) Splenic accumulation of IL-10 mrna in T cells distinct from CD4+CD25+ (Foxp3) regulatory T cells in human visceral leishmaniasis. J Exp Med 204: 805–817. 17389235 10.1084/jem.20061141 PMC2118563

[5] Sacks DL, Lal SL, Shrivastava SN, Blackwell J, Neva FA (1987) An analysis of T cell responsiveness in Indian kala-azar. J Immunol 138: 908–913. 3100620

[6] Hailu A, van der Poll T, Berhe N, Kager PA (2004) Elevated plasma levels of interferon (IFN)-gamma, IFN-gamma inducing cytokines, and IFN-gamma inducible CXC chemokines in visceral leishmaniasis. Am J Trop Med Hyg 71: 561–567. 15569785

[7] Vouldoukis I, Becherel PA, Riveros-Moreno V, Arock M, da Silva O, et al (1997) Interleukin-10 and interleukin-4 inhibit intracellular killing of Leishmania infantum and Leishmania major by human macrophages by decreasing nitric oxide generation. Eur J Immunol 27: 860–865. 9130636 10.1002/eji.1830270409

[8] Gidwani K, Jones S, Kumar R, Boelaert M, Sundar S (2011) Interferon-gamma release assay (modified quantiferon) as a potential marker of infection for Leishmania donovani, a proof of concept study. Plos Negl Trop Dis 5: e1042. 21526219 10.1371/journal.pntd.0001042 PMC3079582

[9] Ansari NA, Kumar R, Gautam S, Nylen S, Singh OP, et al (2011) IL-27 and IL-21 are associated with T cell IL-10 responses in human visceral leishmaniasis. J Immunol 186: 3977–3985. 21357266 10.4049/jimmunol.1003588 PMC3076633

[10] Singh OP, Gidwani K, Kumar R, Nylen S, Jones SL, et al (2012) Reassessment of immune correlates in human visceral leishmaniasis as defined by cytokine release in whole blood. Clin Vaccine Immunol 19: 961–966. 22539471 10.1128/CVI.00143-12 PMC3370446

[11] Gautam S, Kumar R, Maurya R, Nylen S, Ansari N, et al (2011) IL-10 neutralization promotes parasite clearance in splenic aspirate cells from patients with visceral leishmaniasis. J Infect Dis 204: 1134–1137. 21881130 10.1093/infdis/jir461 PMC3164427

[12] Abebe T, Takele Y, Weldegebreal T, Cloke T, Closs E, et al (2013) Arginase activity - a marker of disease status in patients with visceral leishmaniasis in ethiopia. Plos Negl Trop Dis 7: e2134. 23556019 10.1371/journal.pntd.0002134 PMC3610885

[13] Moreno Y, Gros PP, Tam M, Segura M, Valanparambil R, et al (2011) Proteomic analysis of excretory-secretory products of Heligmosomoides polygyrus assessed with next-generation sequencing transcriptomic information. Plos Negl Trop Dis 5: e1370. 22039562 10.1371/journal.pntd.0001370 PMC3201918

[14] Maurya R, Mehrotra S, Prajapati VK, Nylen S, Sacks D, et al (2010) Evaluation of blood agar microtiter plates for culturing leishmania parasites to titrate parasite burden in spleen and peripheral blood of patients with visceral leishmaniasis. J Clin Microbiol 48: 1932–1934. 20335419 10.1128/JCM.01733-09 PMC2863905

[15] Belkaid Y, Mendez S, Lira R, Kadambi N, Milon G, et al (2000) A natural model of Leishmania major infection reveals a prolonged “silent” phase of parasite amplification in the skin before the onset of lesion formation and immunity. J Immunol 165: 969–977. 10878373 10.4049/jimmunol.165.2.969

[16] Murray HW (1990) Effect of continuous administration of interferon-gamma in experimental visceral leishmaniasis. J Infect Dis 161: 992–994. 2157773 10.1093/infdis/161.5.992

[17] Murray HW, Stern JJ, Welte K, Rubin BY, Carriero SM, et al (1987) Experimental visceral leishmaniasis: production of interleukin 2 and interferon-gamma, tissue immune reaction, and response to treatment with interleukin 2 and interferon-gamma. J Immunol 138: 2290–2297. 3104456

[18] Taylor AP, Murray HW (1997) Intracellular antimicrobial activity in the absence of interferon-gamma: effect of interleukin-12 in experimental visceral leishmaniasis in interferon-gamma gene-disrupted mice. J Exp Med 185: 1231–1239. 9104810 10.1084/jem.185.7.1231 PMC2196266

[19] Gautam S, Kumar R, Singh N, Singh AK, Rai M, et al (2013) CD8 T Cell Exhaustion in Human Visceral Leishmaniasis. J Infect Dis 209: 290–299. 23922369 10.1093/infdis/jit401 PMC3873784

[20] Xu Y, mckenna RW, Karandikar NJ, Pildain AJ, Kroft SH (2005) Flow cytometric analysis of monocytes as a tool for distinguishing chronic myelomonocytic leukemia from reactive monocytosis. Am J Clin Pathol 124: 799–806. 16203279 10.1309/HRJ1-XKTD-77J1-UTFM

[21] Fakiola M, Strange A, Cordell HJ, Miller EN, Pirinen M, et al (2013) Common variants in the HLA-DRB1-HLA-DQA1 HLA class II region are associated with susceptibility to visceral leishmaniasis. Nat Genet 45: 208–213. 23291585 10.1038/ng.2518 PMC3664012

[22] Murray HW, Oca MJ, Granger AM, Schreiber RD (1989) Requirement for T cells and effect of lymphokines in successful chemotherapy for an intracellular infection. Experimental visceral leishmaniasis. J Clin Invest 83: 1253–1257. 2539396 10.1172/JCI114009 PMC303815

[23] Kenney RT, Sacks DL, Gam AA, Murray HW, Sundar S (1998) Splenic cytokine responses in Indian kala-azar before and after treatment. J Infect Dis 177: 815–818. 9498473 10.1086/517817

[24] Sundar S, Murray HW (1995) Effect of treatment with interferon-gamma alone in visceral leishmaniasis. J Infect Dis 172: 1627–1629. 7594733 10.1093/infdis/172.6.1627

[25] Sundar S, Singh VP, Sharma S, Makharia MK, Murray HW (1997) Response to interferon-gamma plus pentavalent antimony in Indian visceral leishmaniasis. J Infect Dis 176: 1117–1119. 9333181 10.1086/516526

