# Supplementary figures and images for: Leishmania Specific CD4 T Cells Release IFNγ That Limits Parasite Replication in Patients with Visceral Leishmaniasis
Source: PLoS Negl Trop Dis. 2014 Oct 2;8(10):e3198. doi: 10.1371/journal.pntd.0003198 (PMC4183461; doi:10.1371/journal.pntd.0003198)

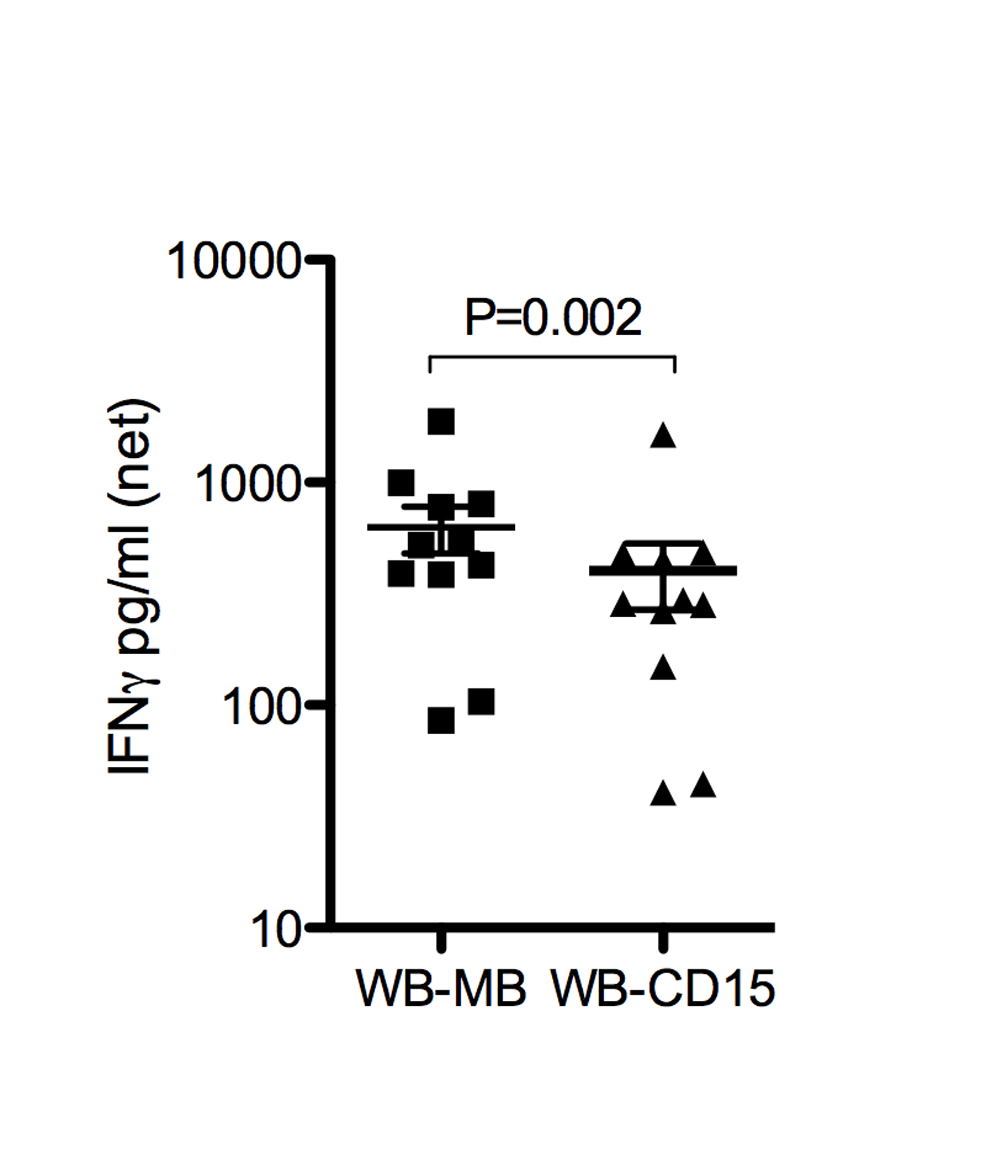

Supplement: Figure S1 — CD15 cells contribute to SLA responses in WB cultures of VL patients. Effect of CD15 depletion (MACS, Miltenyi) on SLA driven IFN-γ response in WB cultures from VL patients. Net responses (SLA stimulated minus unstimulated are shown). Comparison between treatments (CD15 beads or control beads = MB) was made using Wilcoxon matched paired test, and statistical significance is indicated with P-value. (TIF) [file pntd.0003198.s001.tif]
